# Familial Correlations of Onset Age of Hepatocellular Carcinoma: A Population-Based Case-Control Family Study

**DOI:** 10.1371/journal.pone.0108391

**Published:** 2014-09-23

**Authors:** Li Liu, Lixia Li, Shudong Zhou, Qingwu Jiang, Sidong Chen, Yanhui Gao, Yue Chen

**Affiliations:** 1 Department of Epidemiology and Biostatistics and Guangdong Key Lab of Molecular Epidemiology, School of Public Health, Guangdong Pharmaceutical University, Guangzhou, China; 2 Department of Epidemiology, School of Public Health, Fudan University, Shanghai, China; 3 Department of Epidemiology and Community Medicine, Faculty of Medicine, University of Ottawa, Ottawa, Ontario, Canada; MOE Key Laboratory of Environment and Health, School of Public Health, Tongji Medical College, Huazhong University of Science and Technology, China

## Abstract

**Background:**

There was lack of evidence for familial aggregation in onset age of hepatocellular carcinoma (HCC) in Chinese population. We conducted a population-based case-control family study to examine familial correlation of age of HCC onset in Taixing, China.

**Methods:**

A total of 202 cases and 202 matched controls as well as their relatives were included in the study. Lifetime cumulative risks of HCC were estimated using the Kaplan-Meier approach. Cross ratios (CRs) were obtained from stratified Cox proportional hazard models, to assess the familial correlation of onset age.

**Results:**

The mean age of HCC onset was decreased as increasing number of HCC cases in a family. The onset age was the earliest for first-degree relatives, intermediate for second-degree relatives, and latest for non-blood relatives (spouse) (log-rank test, *P*<0.01). The onset age was significantly correlated between probands and their relatives. In stratified Cox proportional hazard models, the CRs for the probands versus their fathers, mothers, siblings and uncles/aunts were 6.25 (95% confidence interval (CI): 1.84–21.25), 9.81 (95% CI: 1.24–77.56), 6.22 (95% CI: 1.37–28.36) and 3.24 (95% CI: 1.26–8.33), respectively. After adjustment for hepatitis B virus infection, the CRs remained significant.

**Conclusion:**

This current study suggested a significant correlation of onset age for HCC among blood relatives. Familial HCC cases yielded earlier age of onset and their relatives have higher HCC risk in early age, highlighting intensive surveillance should be start at an earlier age for individuals with family history of HCC.

## Introduction

Hepatocellular carcinoma (HCC) is the fifth most frequent cancer and ranks the third leading cause of cancer mortality in the world [1]. Half of newly diagnosed HCC cases (approximately 400,000 cases) and deaths were estimated to occur in China [1]. Hepato-carcinogenesis is a complicated process driven by diverse etiologies involving in both environmental and genetic factors [2]. The most important risk factor for HCC is hepatitis B (HBV) exposure, with more than 350 million infected individuals worldwide [3]. In the epidemic area such as China, HBV infection accounts for approximately 80% of HCC cases [3]. Other well-recognized risk factors are heavy alcohol drinking, aflatoxin exposure, and contaminated drinking water [2].

Strong familial aggregation for HCC has been frequently observed [4], [5], [6], [7]. In Eastern countries, HBV transmission among family members might be responsible for part of the observed familial aggregation [4], while in Western population, a family history of HCC increases HCC risk independently of hepatitis [7]. Variability in onset age of HCC has been also noted in these studies. The peak onset’ age varies according to geographical areas [3], where in China the mean age of diagnosis with HCC was 55–59 years and 63–65 years in Europe. More importantly, the study of 5080 patients in Hong Kong reported a lower average age of onset in familial patients (48.5±13 years) than those in sporadic patients (62±11 years) [8]. A similar phenomenon was suggested by a recent study in Korea [6]. However, inconsistent results have been reported that onset age in patients with family history and in those without family history did not differ significantly [9], [10]. So far, there was a lack of evidence for familial association in onset age of HCC in Mainland China. Herein, we conducted a population-based case-control family study, including 202 cases and 202 matched controls as well as their relatives from Taixing, China, to examine the familial correlation of age of HCC onset.

## Materials and Methods

### Proband selection and data collection

We conducted a population-based case-control family study of hepatocellular carcinoma and the details of study design and data collection had been reported elsewhere [4]. Briefly, this study was conducted in Taixing, Jiangsu province, China, between 2001 and 2002. All case probands who were newly diagnosed with HCC between 2000 and 2002, were ascertained through a surveillance system at the Center for Disease Control and Prevention in Taixing (n = 202). Control probands were randomly selected from a government register of all Taixing residents and 1:1 matched by age (age at diagnosis for case probands) (±3 years), sex and county residence. Controls who had kinship with matched case were excluded. For all probands, their non-blood relatives (spouses) and first-degree (natural parents and full siblings) and second-degree (paternal and maternal uncles and aunts) relatives were identified, including a total of 1503 relatives of cases and 1433 relatives of controls after excluding those who had missing age, younger than 20 years or had died before the age of 20 years.

Ascertained families were interviewed by well-trained research assistants. Information on disease status and onset age was collected for the relatives of both cases and controls, and was treated as a composite disease outcome. If subjects had liver cancer at the time of study, onset age was the age at diagnosis for HCC subjects; otherwise, last known age was considered as censoring onset age for non-HCC subjects. Data on risk factors included sex, smoking history (yes, no), drinking history (yes, no), main source of drinking water (ditch water, river, well and tap water), main staple food (rice, wheat and corn) and hepatitis B surface antigen (HBsAg) (positive, negative and unknown). In this study, a person who smoked at least one cigarette per day for one year or more was defined as a smoker. A drinker was defined as a person who drank alcohol at least once a week for one year or more [4]. Because several HBV-related screening studies were conducted in the population before our investigation, for HBsAg status of all participants were asked for showing previously screening records if possible. At recruitment, written informed consent was obtained from each subject. This study was conducted under the approval of the institutional review board of Guangdong Pharmaceutical University.

### Statistical analysis

The Kaplan-Meier survival analysis was conducted to estimate the lifetime cumulative risk of HCC for the relatives of cases and controls as well as for case families stratified by type of relatives (first-degree relatives, second-degree relatives, no blood relatives). A log-rank test was used to test differences between the survival curves.

Cross ratio (CR) function [11] was used to assess the familial correlation of onset age for HCC. The null value for CR is 1 and a larger value indicates a stronger dependence of onset age for HCC between two members. CR was estimated using the approach of stratified Cox proportional hazard model [12], [13]. Model parameters were estimated using the method of maximum partial likelihood. All models are stratified by age of the probands’ (<50 and ≥50 years). We accounted for the non-independence of observations within families by using a robust variance estimate [14]. All analyses were carried out using the IBM SPSS statistics software version 21.0.

## Results

The baseline characteristics of participants have been described in our previous epidemiologcial study [4]. In brief, The mean age of HCC onset was 52.4 (±11.4) years for 202 case probands. The minimum and maximum onset age were 26 and 80 years old, respectively. A total of 87 HCC cases were reported among the relatives, including 71 from 202 case families and 16 from 202 control families. The mean age of onset was decreased with increasing number of HCC cases in a family. In case families with 1, 2 and 3 affected member(s), the mean onset age were 58.6 (±13.6, n = 38), 53.4 (±11.5, n = 9) and 48.7 (±12.5, n = 5) years, respectively. Approximately 75% of cases were males. Positive HBsAg was reported among 38.0% of affected case relatives, and 18.8% of affected control relatives compared with 5.4% and 1.0% of their unaffected counterparts, respectively.

**Figure 1:**
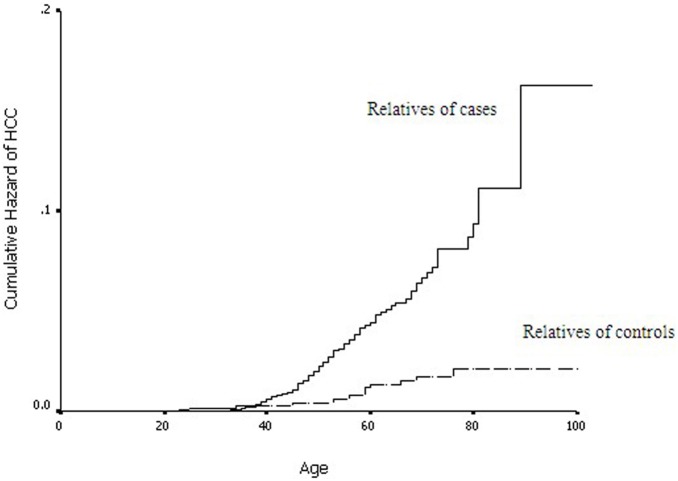
Kaplan-Meier curves of the lifetime cumulative hazard of HCC for relatives of cases and controls (log-rank test, *P*<0.001).

**Figure 2:**
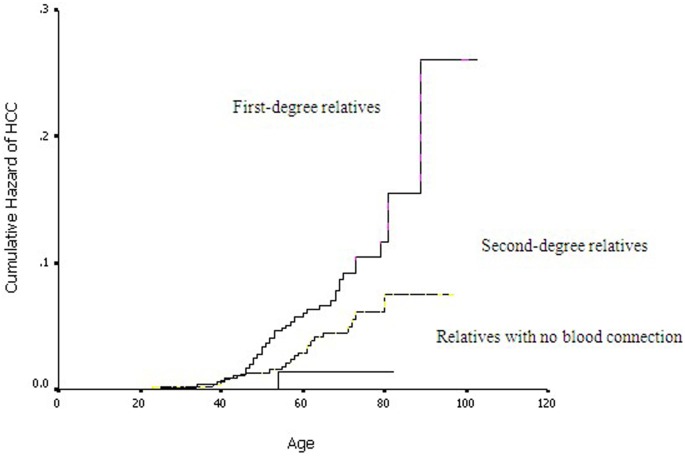
Kaplan-Meier curves of the lifetime cumulative hazard of HCC for relatives of cases by relationship type (log-rank test, *P*<0.001).

Figure 1 shows Kaplan-Meier curves of the lifetime cumulative hazard of liver cancer for relatives of cases and controls. Starting at the age of 40 years, the cumulative risk of liver cancer increased more quickly and the onset age was significantly younger in case relatives than control relatives (log-rank test, *P*<0.001). Figure 2 shows the lifetime cumulative hazard of HCC for case relatives by kinship. The age of onset was the earliest for first-degree relatives, intermediate for second-degree relatives, and latest for non-blood relatives (spouse) (log-rank test, *P*<0.001).

Table 1 presents the CRs for onset age of HCC by relationship type after adjustment for sex and proband’s age. The CRs for all types of blood relatives including mothers, fathers, siblings and uncles/aunts, were significantly larger than 1 and the CR for spouses was not significantly different from a unit, suggesting an increased risk for relatives of earlier onset cases.

**Table 1:** Familial correlation of age at onset for HCC by type of relationship to probands in stratified Cox proportional hazard models.

Relationship Type	Sex (male/female)		Age of probands		Familial correlation	
	*β (SE)*	*P*	*β (SE)*	*P*	CR (95% CI)	*P*
Fathers			–0.09 (0.04)	0.022	6.25 (1.84,21.25)	0.003
Mothers			–0.03 (0.05)	0.563	9.81 (1.24,77.56)	0.03
Siblings	2.18 (0.76)	0.004	–0.09 (0.05)	0.042	6.22 (1.37,28.36)	0.018
Uncles/aunts	0.46 (0.42)	0.272	–0.02 (0.05)	0.749	3.24 (1.26,8.33)	0.015
Spouses	0.59 (1.24)	0.637	0.02 (0.07)	0.786	0.33 (0.03,3.42)	0.354

To determine whether the familial association for the onset age of HCC was influenced by common risk factors such as contaminated drinking water and HBV infection, we also analyzed data including all relatives (Table 2). Model 1 showed that the crude familial correlation of onset age was significant (CR = 4.4, *P*<0.001). Model 2 showed river water and tap water had significant protective effects on HCC. However, drinking water had little effect on the familial correlation of onset age for HCC (likelihood ratio test, *χ^2^* = 4.43, *P* = 0.35). When HBV infection was taken into consideration, the familial correlation reduced but remained significant (CR = 2.94, *P*<0.001; likelihood ratio test, *χ^2^* = 52.89, *P*<0.001), indicating that the common environmental exposures did not explain all the familial correlation in onset age of HCC.

**Table 2:** Familial correlation of onset age for HCC adjusted by HbsAg status and source of drinking water.

Variable	Model 1		Model 2		Model 3	
	β (SE)	*P*	β (SE)	*P*	β (SE)	*P*
male/female	0.92 (0.24)	<0.001	0.84 (0.24)	<0.001	0.76 (0.25)	0.002
Age of probands	–0.04 (0.02)	0.092	–0.04 (0.02)	0.075	–0.02 (0.02)	0.117
Drinking water*						
Ditch			0.19 (0.43)	0.65	0.26 (0.38)	0.499
River			–0.54 (0.25)	0.031	–0.66 (0.24)	0.006
Well			–0.66 (0.34)	0.055	–0.64 (0.34)	0.062
Tap			–0.72 (0.30)	0.016	–0.65 (0.28)	0.020
HbsAg status						
Positive					4.32 (0.74)	<0.001
Unknown					1.59 (0.75)	0.034
CR (95% CI)	4.39(2.45,7.87)	<0.001	4.26(2.36,7.68)	<0.001	2.94(1.59,5.45)	0.001
–2logL	1104.99		1087.26		981.49	
No. of parameters	3		7		9	
Models compared			2 vs. 1		3 vs. 2	
LR test			4.43		52.89	
df			4		2	
*P*-value			0.35		<0.001	

Abbreviations: CR, cross ratio; 95% CI, 95% confidence interval; LR, Likelihood ratio.

*Four types of drinking water were adjusted for each other.

## Discussion

For most chronic diseases, an individual who is absence of a disease at a certain time would develop that disease later in life. When assessing the familial aggregation of disease, simple classification of disease outcome as a dichotomous variable (yes/no), with ignoring the information on onset age and bringing in a censoring effect, would lead to a biased estimate and/or lose efficiency [15]. Therefore, it is important for analysis of familial aggregation to capture not only the correlation of disease incidence but also the correlation of onset ages among relatives [12]. In this population-based case-control family study, we suggested a younger average age of onset among cases with familial HCC and a correlation of onset age between probands and their relatives.

HBV infection plays a major carcinogenic role in global HCC epidemiology. HBV spread within family has been thought to one reason why family history of HCC is a risk factor [8]. However, Lok and Lai [16] found that the cluster HCC in families could not explained by a higher HBsAg carrier rate or earlier ages of the individuals the virus infect. A recent study in the United States showed family history was associated with a 4.1-fold increased risk of HCC (95% CI 1.3–12.9) in the individuals without viral hepatitis [9]. In this study, after adjustment for HBV infection, the CRs for familial correlation of onset age was reduced, but retained significant, suggesting other risk factors would be involved in the family aggregation of HCC. It has been reported that heavy alcohol intake might also accelerate the onset of HCC [17]. Alcohol has been suggested as an important risk factor for HCC in developed countries [2]. However, no association between alcohol drinking and HCC was found in this Chinese population.

For some other complex disease such as familial breast cancer and Alzheimer’s disease, the early-onset cases might have strong genetic component [18], [19]. In other words, carriers of high-risk alleles are more likely to have an early-onset disease as compared with non-carriers. Intriguingly, in this study, onset age for HCC was significantly earlier in relatives of cases than those of controls, and average age of onset was inversely associated with the number of relatives with HCC. The lifetime cumulative risk of HCC in case relatives was increased with increasing blood connection with probands. Furthermore, the familial correlation of onset age was greater for those with their blood relationship closer to the probands (null between husbands and wives, moderate between the probands and their uncles/aunts, and strong between parents and offspring or between sibling pairs), suggesting a possibility of genetic additive variance. Taken together, these observations supported the significant contribution of genetic factors in family aggregation of HCC. Multiple genetic variations have been found in previous studies to influence onset age of HCC, such as variations in *IL28B* gene, P53 pathway, and TGF-beta signaling pathway [20], [21], [22]. A recent study in a Chinese population suggested genetic background of killer cell immunoglobulin-like receptor (*KIR*) and human leukocyte antigen (*HLA*) genes could influence the onset age of HCC in males with HBV infection [23]. Nevertheless, the effects of genetic factors in familial clustering of HCC remain to be further evaluated.

In this current study, a stratified Cox model allows cross ratio to be piecewise constant over the elements of a grid formed by the proband and relative ages [13]. However, limited by sample size, more age groups would lead to inefficient and unstable estimators [12]. Additionally, many of parents, uncles and aunts of probands had died when this investigation was conducted, which was a main reason for missing HbsAg. We had a separate group for those with missing HbsAg in modelling. When we compared the HbsAg(–) group, the relative risk for HbsAg-unknown group was greater than unity but the effect is less than the HbsAg(+) group. Another potential limitation was that case subjects were more likely to be aware of a family history of cancer than control subjects. Nevertheless, Taixing is a region with high incidences of both HCC and HBV infection, and several HBV-related screening studies have been conducted in the population, which increased awareness of these conditions in the whole population including case and non-case families.

## Conclusion

This population-based case-control family study showed a significant familial correlation of onset age of hepatocellular carcinoma between probands and their relatives. Familial HCC cases have earlier age of onset and their relatives have a higher HCC risk in an early age, highlighting intensive surveillance should be start at an earlier age for individuals with family history of HCC.
